# Interstitial Cystitis Can Be Improved With Intravesical Instillation of Platelet-Rich Plasma

**DOI:** 10.7759/cureus.22747

**Published:** 2022-03-01

**Authors:** Timothy J Hardy

**Affiliations:** 1 Gynecology, Bon Secours Maryview Medical Center, Portsmouth, USA

**Keywords:** intravesical instillation, painful bladder syndrome, hydrodistention, plasma-rich platelets, interstitial cystitis

## Abstract

Intravesical instillation of platelet-rich plasma has the potential to improve symptoms and reduce pain in patients who have interstitial cystitis and painful bladder syndrome by utilizing the body's own growth factors found in platelets. Interstitial cystitis is a disease of the bladder that causes pain, urinary frequency, urgency, and nocturia. It is difficult to treat and has unknown etiology. Patients who have interstitial cystitis have high rates of anxiety as a comorbid condition. People with anxiety are known to have dysregulation of serotonin. These concepts are interrelated.

## Introduction

Orthopedic literature has many studies showing the benefits of platelet-rich plasma (PRP). When injected into the intra-articular joints and tendons, the growth factors help with repair. A previously published study from China showed that PRP was effective through injection in multiple areas of the bladder over multiple treatment cycles [1]. I present a case study utilizing platelet-rich plasma during hydrodistention for patients with chronic interstitial cystitis unresponsive to current therapies.

## Case presentation

A 45-year-old female patient, gravida 3 para 1, presented with a greater than 10-year history of interstitial cystitis that had been treated with pentosan polysulfate (pentosan polysulfate sodium; ELMIRON), anticholinergic medication, and intravesical heparin instillation, all with inconsistent results. The patient had also undergone hysterectomy for endometriosis and was being treated with an antidepressant for depression. 

**Figure 1:**
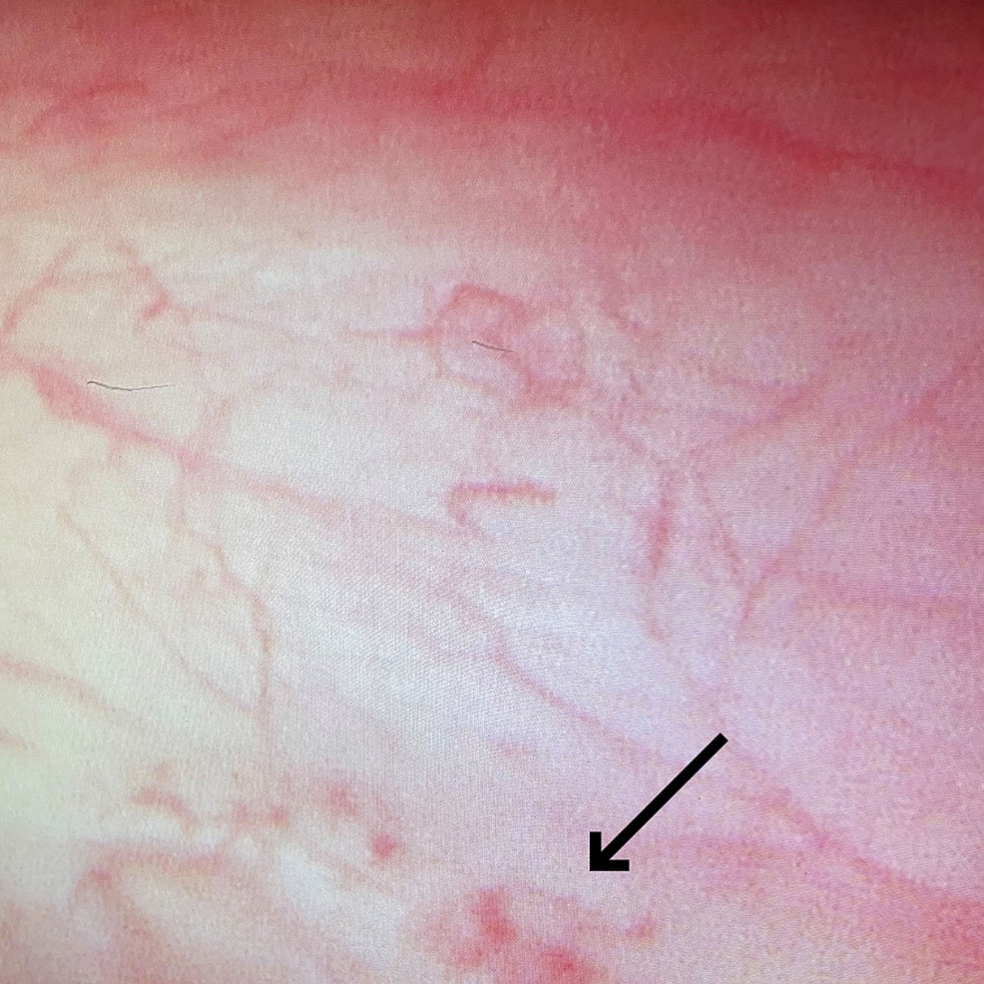
Cystoscopy on patient showing patchy erythema (arrow)

Under conscious sedation, cystoscopy was performed which showed slight erythema of the urothelium and squamous metaplasia of the trigone with no other focal lesions (Figure 1). The bladder was distended with 700 mL of normal saline which was the maximum cystometric bladder capacity, with 80 cm of water pressure by gravity. After three minutes dwell time the saline was removed. Mucosal bleeding after distention was noted. 50 mL of blood from the patient was removed from a peripheral vein before the procedure and separated. 30 mL of platelet-rich plasma was infused into the bladder, and the patient maintained it in the bladder for two hours. 

**Figure 2:**
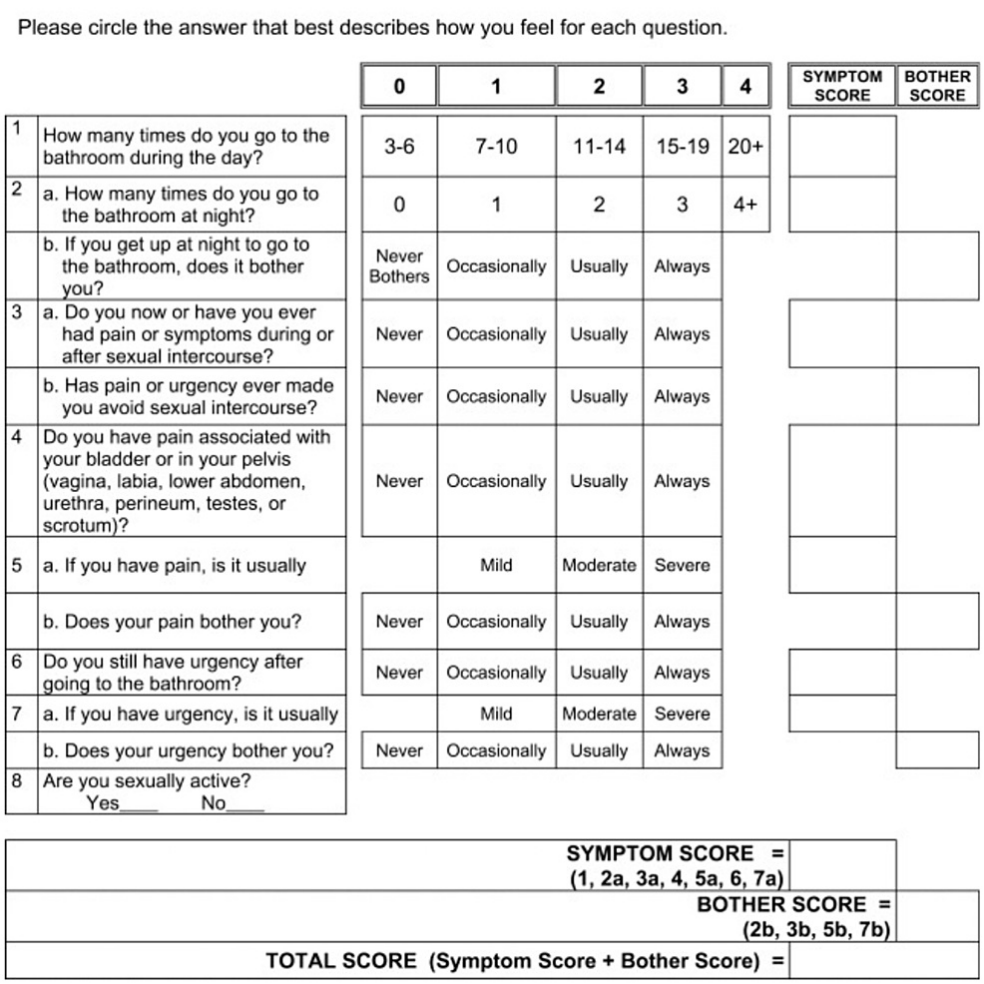
Pelvic Pain and Urgency/Frequency Patient Symptom Scale

On follow-up, six weeks after initial treatment, there was an improvement of her Pelvic Pain and Urgency/Frequency patient symptom scale score (Figure 2) [2]. She noticed decreased urinary frequency, urgency, and nocturia. She had a decrease in her total score (symptom score + bother score) from 20 to 2. She was voiding three to six times per day and once at night. She denied pain with a full bladder or any urgency symptoms.

## Discussion

The hypothalamic-pituitary-adrenal axis maintains homeostasis in emergency, or “fight or flight,” situations. The autonomic nervous system, involving the sympathetic and parasympathetic nerves, connects the brain to the body. The adrenal response to stressors is closely tied to adrenal cortical hormonal substances, such as norepinephrine and epinephrine. An excessive or prolonged stress response, such as in patients with generalized anxiety disorder and other mental disorders, can contribute to multiple clinical disorders, including interstitial cystitis. This concept was further defined by Goldstein and McEwan in 2002 to describe a more nuanced way of responding to different stressors such as cold, trauma, hemorrhage, and perceived threats in the environment [3]. There is an association between norepinephrine and active escape avoidance, or attack, and an association between epinephrine and passive, immobile fear. As the body repairs itself, growth factors are closely related to the regulation of cell growth and repair. Large amounts of growth factors are in platelets and are released during the healing process.

I am proposing that there is dysregulation of the release of growth factors in individuals who are chronically stressed to try to conserve energy for perceived threats. During stress it is known that blood is shunted away from the vital organs, such as the liver, the bowel, and the brain, and towards muscles in the extremities. There is an increased heart rate and dilation of the coronary blood vessels which increases blood flow and increases availability of oxygen to the heart. There is dilation of blood vessels of skeletal muscles and constriction of blood vessels serving digestion, which increases the availability of oxygen to skeletal muscle and decreases blood flow to the small and large bowel. There is dilation of the bronchi in the lung with increased respiration rate to increase availability of oxygen in the blood. There is increased conversion of glycogen to glucose, which increases availability of glucose to skeletal muscle and brain cells. The skin becomes pale because of constriction of the blood vessels and reduced blood flow to conserve energy for fight or flight. The pupils of the eyes dilate to allow more light into the eyes so the visual acuity is improved to scan nearby surroundings for threats [3].

Autologous platelet-rich plasma takes the person’s own blood and concentrates the plasma by filtration and centrifugation, taking advantage of the known different densities of red blood cells, platelets, lymphocytes, and neutrophils. The goal is to extract the buffy coat and platelet-rich plasma that contains the platelets and concentrate lymphocytes, monocytes, and neutrophils and exclude red blood cells. The blood can be centrifuged, separating the red blood cells into plasma-rich platelets. Each separation tube is proprietary with multiple options that provide different PRP properties and biologic formulations. There is a lack of consensus on standardization preparation protocols. This leads to inconsistent outcomes making it difficult to compare different studies; however, many studies have shown benefits of PRP in the fields of dentistry and orthopedics, specifically in treatment of osteoarthritis and tendonitis [4].

The current science behind platelet-rich plasma preparations is to concentrate the body’s own growth factors and cells required for regeneration and place them in the part of the body that needs healing. Platelets contain many growth factors. Vascular endothelial growth factor increases angiogenesis and vessel permeability and stimulates biogenesis for endothelial cells. Insulin growth factor is a chemotaxis agent for fibroblasts and stimulates protein synthesis. Hepatocyte growth factor regulates cell growth and motility in epithelial and endothelial cells, supporting epithelial repair and neovascularization during wound healing. Keratinocyte growth factor regulates epithelial migration and proliferation. Epidermal growth factor encourages proliferation of keratinocytes and fibroblasts and stimulates mitogenesis for endothelial cells. Angiopoietin induces angiogenesis, stimulates migration and proliferation of endothelial cells, and supports and stabilizes blood vessel development via the recruitment of pericytes. Additionally it is known that leukocytes play an important role in tissue remodeling and repair and are intimately involved with the innate and adaptive immune response to foreign bodies [4].

Stressful conditions and general anxiety cause a relative deficiency in serotonin. Serotonin has been shown to help orchestrate function of bodily organs, including the bladder and bowel [5]. When epithelial cell integrity breaks down, there is a decreased production of the protective mucous layer, allowing toxic solutes to enter the epithelial and subepithelial bladder mucosa and initiate an inflammatory response, including mast cell activation [6].

Platelet granules have a high level of serotonin, which has a well-defined role in the central nervous system and in the body to regulate perception of pain and also influences inflammation in the body [4]. In the central nervous system serotonin is decreased in patients with anxiety and depression and can be modulated by selective serotonin receptor inhibitors to increase serotonin. In addition to improving anxiety and depression, SSRIs can also improve pain tolerance for chronic pain conditions [5]. The majority of serotonin in the body is found outside the central nervous system where it regulates systemic and cellular biologic functions in many organs, including the gastrointestinal, genitourinary, pulmonary, and cardiovascular systems. Serotonin can influence adipocytes, epithelial cells, and leukocytes in a dose-dependent relationship. Serotonin in the periphery of the body is an immune modulator that affects inflammation. It can also affect other immune cells through their serotonin receptors. It is postulated that release of serotonin and other growth factors from platelet granules has an analgesic effect through serotonin receptors. During platelet activation, platelets develop pseudopods which help with platelet aggregation. They also release their intracellular alpha and dense granules which release growth factors cytokines and serotonin. Using plasma-rich platelets increases the local concentration of serotonin and growth factors in the targeted tissue [4].

Interstitial cystitis (IC) is a chronic pain condition of unknown etiology that includes symptoms of urinary frequency, urgency, and pain perceived to originate from the urinary bladder [7]. Many patients who suffer from interstitial cystitis also suffer from many other disorders, including anxiety. The prevailing theory on the etiology of interstitial cystitis is alteration in the urothelium [8]. A defect in the glycosaminoglycan layer of the urothelium allows toxic urinary products to invade the urinary epithelium and initiate inflammatory response and mast cell activation [7]. Most patients report worsening of their condition with stressful circumstances [9].

Stress can cause permanent changes in the sympathetic division of the autonomic nervous system with reduction in descending inhibition causing increase in bladder pain sensation, also termed stress-induced hyperalgesia. A study showed that IC patients exhibit increased sympathetic function and decreased vagal activity as shown by decreased heart rate variability [5]. 

I am proposing that during this process of chronic stress certain parts of the body, such as the urothelium, do not undergo repair. When the body perceives chronic stress, it is deferring maintenance in preparation for a “life or death” event. This decreases blood flow and decreases availability of growth factors. When maintenance is deferred, repeated trauma causes an increase in inflammation and a defect in the urothelium.

Stressful conditions and general anxiety cause a relative deficiency in serotonin. Serotonin has been shown to help orchestrate function of bodily organs, including the bladder and bowel [5]. When epithelial cell integrity breaks down, there is a decreased production of the protective mucous layer, allowing toxic solutes to enter the epithelial and subepithelial bladder mucosa and initiate an inflammatory response, including mast cell activation [6].

## Conclusions

During hydrodistention the thin urothelium cracks, and the subepithelial layers are exposed. The growth factors in the plasma-rich platelets facilitate healing. By utilizing PRP in conjunction with hydrodistention, it allows for a global application method for treatment of the bladder for patients who have interstitial cystitis. This preliminary study requires further evaluation to confirm the usefulness of PRP in patients with interstitial cystitis and to determine the duration of the effect. The conclusion is valid only for this individual case report, and more controlled studies are needed in the future to confirm these findings. 
